# Maternal Serum Metabolomics in Mid-Pregnancy Identifies Lipid Pathways as a Key Link to Offspring Obesity in Early Childhood

**DOI:** 10.3390/ijms25147620

**Published:** 2024-07-11

**Authors:** Ellen C. Francis, Katerina Kechris, Randi K. Johnson, Shristi Rawal, Wimal Pathmasiri, Blake R. Rushing, Xiuxia Du, Thomas Jansson, Dana Dabelea, Susan J. Sumner, Wei Perng

**Affiliations:** 1 Department of Biostatistics & Epidemiology, Rutgers School of Public Health, Piscataway, NJ 08854, USA; 2 Department of Biostatistics & Informatics, Colorado School of Public Health, Aurora, CO 80045, USA; katerina.kechris@cuanschutz.edu; 3 Department of Biomedical Informatics, University of Colorado Anschutz Medical Campus, Aurora, CO 80045, USA; randi.johnson@cuanschutz.edu; 4 Department of Epidemiology, Colorado School of Public Health, University of Colorado Anschutz Medical Campus, Aurora, CO 80045, USA; dana.dabelea@cuanschutz.edu (D.D.); wei.perng@cuanschutz.edu (W.P.); 5 Department of Clinical and Preventive Nutrition Sciences, School of Health Professions, Rutgers University, Newark, NJ 07102, USA; shristi.rawal@rutgers.edu; 6 Department of Nutrition, Gillings School of Global Public Health, University of North Carolina at Chapel Hill, Chapel Hill, NC 27599, USA; wimal_pathmasiri@unc.edu (W.P.); blake_rushing@unc.edu (B.R.R.); 7 Nutrition Research Institute, University of North Carolina at Chapel Hill, Chapel Hill, NC 27599, USA; 8 Department of Bioinformatics and Genomics, University of North Carolina at Charlotte, 9201 University City Blvd, Charlotte, NC 28223, USA; xiuxia.du@charlotte.edu; 9 Department of Obstetrics and Gynecology, University of Colorado Anschutz Medical Campus, Aurora, CO 80045, USA; thomas.jansson@cuanschutz.edu; 10 The Lifecourse Epidemiology of Adiposity and Diabetes (LEAD) Center, University of Colorado Anschutz Medical Campus, Aurora, CO 80045, USA

**Keywords:** pregnancy, gestational diabetes mellitus, metabolomics, lipids, WGCNA

## Abstract

Maternal metabolism during pregnancy shapes offspring health via in utero programming. In the Healthy Start study, we identified five subgroups of pregnant women based on conventional metabolic biomarkers: Reference (*n* = 360); High HDL-C (*n* = 289); Dyslipidemic–High TG (*n* = 149); Dyslipidemic–High FFA (*n* = 180); Insulin Resistant (IR)–Hyperglycemic (*n* = 87). These subgroups not only captured metabolic heterogeneity among pregnant participants but were also associated with offspring obesity in early childhood, even among women without obesity or diabetes. Here, we utilize metabolomics data to enrich characterization of the metabolic subgroups and identify key compounds driving between-group differences. We analyzed fasting blood samples from 1065 pregnant women at 18 gestational weeks using untargeted metabolomics. We used weighted gene correlation network analysis (WGCNA) to derive a global network based on the Reference subgroup and characterized distinct metabolite modules representative of the different metabolomic profiles. We used the mummichog algorithm for pathway enrichment and identified key compounds that differed across the subgroups. Eight metabolite modules representing pathways such as the carnitine–acylcarnitine translocase system, fatty acid biosynthesis and activation, and glycerophospholipid metabolism were identified. A module that included 189 compounds related to DHA peroxidation, oxidative stress, and sex hormone biosynthesis was elevated in the Insulin Resistant–Hyperglycemic vs. the Reference subgroup. This module was positively correlated with total cholesterol (R:0.10; *p*-value < 0.0001) and free fatty acids (R:0.07; *p*-value < 0.05). Oxidative stress and inflammatory pathways may underlie insulin resistance during pregnancy, even below clinical diabetes thresholds. These findings highlight potential therapeutic targets and strategies for pregnancy risk stratification and reveal mechanisms underlying the developmental origins of metabolic disease risk.

## 1. Introduction

Pregnancy is associated with profound metabolic changes that are critical for normal fetal development. With rising rates of obesity worldwide, developmental overnutrition or exposure of the fetus to excess fuels (e.g., glucose, insulin, free fatty acids), is a major concern as it increases offspring risk of excess adiposity and metabolic risk across their life course [1,2,3,4,5]. Maternal metabolic profiles, specifically those associated with maternal overweight/obesity and gestational diabetes mellitus (GDM), are two very common conditions exposing the fetus to overnutrition, that affects the fetus indirectly through changes in placental function [6] or directly by altering the in utero metabolic milieu [7,8]. Currently 29% of women in the United States have pre-pregnancy obesity [9], and 8% of pregnancies are impacted by GDM [10]. 

Many placental and maternal endocrine compounds contribute to the prenatal metabolic milieu and include factors such as maternal insulin–glucose metabolism, triglyceride levels, and free fatty acids [11,12,13]. Our prior work in the Healthy Start study, investigating a large pre-birth cohort of general-risk pregnant women and their offspring [14], utilized seven conventional metabolic biomarkers assayed from mid-pregnancy fasting blood plus two derived indices to capture metabolic heterogeneity in pregnancy [15]. Using these data, we identified five metabolic subgroups among pregnant participants: High High-Density Lipoprotein Cholesterol (HDL-C), Dyslipidemic–High Triglyceride (TG), Dyslipidemic–High Free Fatty Acid (FFA), Insulin Resistant (IR)–Hyperglycemic, and a Reference group—the majority of whom had biomarker levels below clinically-recognized thresholds for metabolic risk [15]. Importantly, these subgroups were differentially associated with offspring outcomes, with the IR–Hyperglycemic and Dyslipidemic–High FFA subgroups showing stronger associations with offspring risk of obesity during early childhood than maternal obesity or GDM. Specifically, the IR–Hyperglycemic subgroup, characterized by insulin resistance and an atherogenic lipid profile (high TGs, high FFAs, low HDL-C) in mid-pregnancy, could lead to a greater fat accrual in offspring through enhanced nutrient transfer and metabolic programming [7]. Maternal hyperglycemia has been reported to be associated with increased adiposity in offspring, possibly due to elevated fetal insulin levels promoting fat storage [16]. Furthermore, the statistical analysis revealed that the odds ratios for childhood obesity were significantly higher in the IR–Hyperglycemic and Dyslipidemic–High FFA subgroups compared to the Reference subgroup, even after adjusting for maternal BMI and gestational weight gain. These findings underscore the need to consider metabolic heterogeneity in pregnancy, as focusing solely on maternal obesity or diabetes may overlook critical pathways influencing offspring metabolic health [15].

Recent advancements in high-throughput technologies have enabled researchers to study thousands of analytes concomitantly, providing a comprehensive overview of physiologic and metabolic processes [17]. In particular, untargeted metabolomics profiling, which assesses the relative abundance of low-molecular weight compounds in a biological sample, is a powerful tool for identifying metabolic pathways that may capture nuanced variation in metabolism than is possible by more traditional single biomarker assays or approaches such as BMI classification. To date, a handful of studies have utilized metabolomics profiling to harness metabolic variation related to common pregnancy complications, such as GDM, preeclampsia, and preterm delivery, providing insight into pathophysiological mechanisms and potential novel therapeutic targets [18,19,20,21,22,23]. However, focusing solely on women diagnosed with a pregnancy complication may limit our ability to gain insight into the potential pathways underlying different adaptations to pregnancy that could adversely influence offspring health even when maternal metabolic markers do not meet the clinical diagnostic thresholds for disease.

Here, our study extends current knowledge, including our own prior study that characterized the five metabolic subgroups of pregnant women [15], by leveraging mid-pregnancy maternal serum metabolomics profiling data to further refine the current understanding of pathways underlying developmental overnutrition. To do this, we conducted a comprehensive analysis using untargeted metabolomics data to identify metabolic pathways associated with each of the five previously identified maternal metabolic subgroups in the Healthy Start pre-birth cohort [14]. We expect that women in the IR–Hyperglycemic subgroup have different metabolomic profiles than the Reference group, particularly for the profiles related to insulin resistance and high levels of circulating lipids. We further expect that metabolomic variation accounts for some of the differences between subgroups and will identify specific pathways related to insulin resistance and dyslipidemia that contribute to differences in the conventional biomarkers that characterized these five subgroups.

In this cohort of 1065 pregnant women, we identified eight metabolomic profiles that represented different pathways such as the carnitine–acylcarnitine translocase system, fatty acid biosynthesis and activation, and glycerophospholipid metabolism. One profile, characterized by DHA peroxidation, oxidative stress, and sex hormone biosynthesis metabolites, differed between the Reference and IR–Hyperglycemic group. These findings highlight that oxidative stress and inflammatory pathways may underlie development of insulin resistance and hyperglycemia during pregnancy, even below clinical thresholds for diabetes.

## 2. Results

### 2.1. Participants

The mean (SD) maternal age at enrollment was 28 (6) years; 23% identified as Hispanic, 15% as non-Hispanic Black, 6% as non-Hispanic Other, and 56% as White. Forty-seven percent attained a college or graduate degree, 19% had obesity prior to pregnancy, 47% experienced excessive weight gain in pregnancy, and 4% were diagnosed with GDM (Table 1). At a mean (SD) of 18 (3) gestational weeks, 2% of women had glucose concentrations above cut-offs for elevated glucose and 31% had TC levels above cut-offs indicative of atherogenic dyslipidemia.

As previously observed [15], we noted differences in background characteristics across the five metabolic subgroups, with the Reference group generally having a lower proportion of perinatal risk factors (e.g., smoking, GDM diagnosis, excessive gestational weight gain). The High HDL-C subgroup had the lowest prevalence of pre-pregnancy obesity (7%) and none had HDL-C ≤ 50 mg/dL. Most (90%) of the Dyslipidemic–High TG group had TGs ≥150 mg/dL. Most (82%) women classified to the Dyslipidemic–High FFA group had FFAs >75th percentile in the full sample. All women in the IR–Hyperglycemic subgroup had HOMA-IR ≥ 2.9, >33% had atherogenic lipid levels (TGs ≥ 150 mg/dL, HDL-C ≤ 50 mg/dL) [19], 55% had excessive GWG, and 59% had pre-pregnancy obesity; this subgroup also had the highest prevalence of GDM (17%), lowest prevalence of nulliparity (40%), and youngest age.

### 2.2. Maternal Mid-Pregnancy Metabolomic Profiling Modules

**Figure 1:**
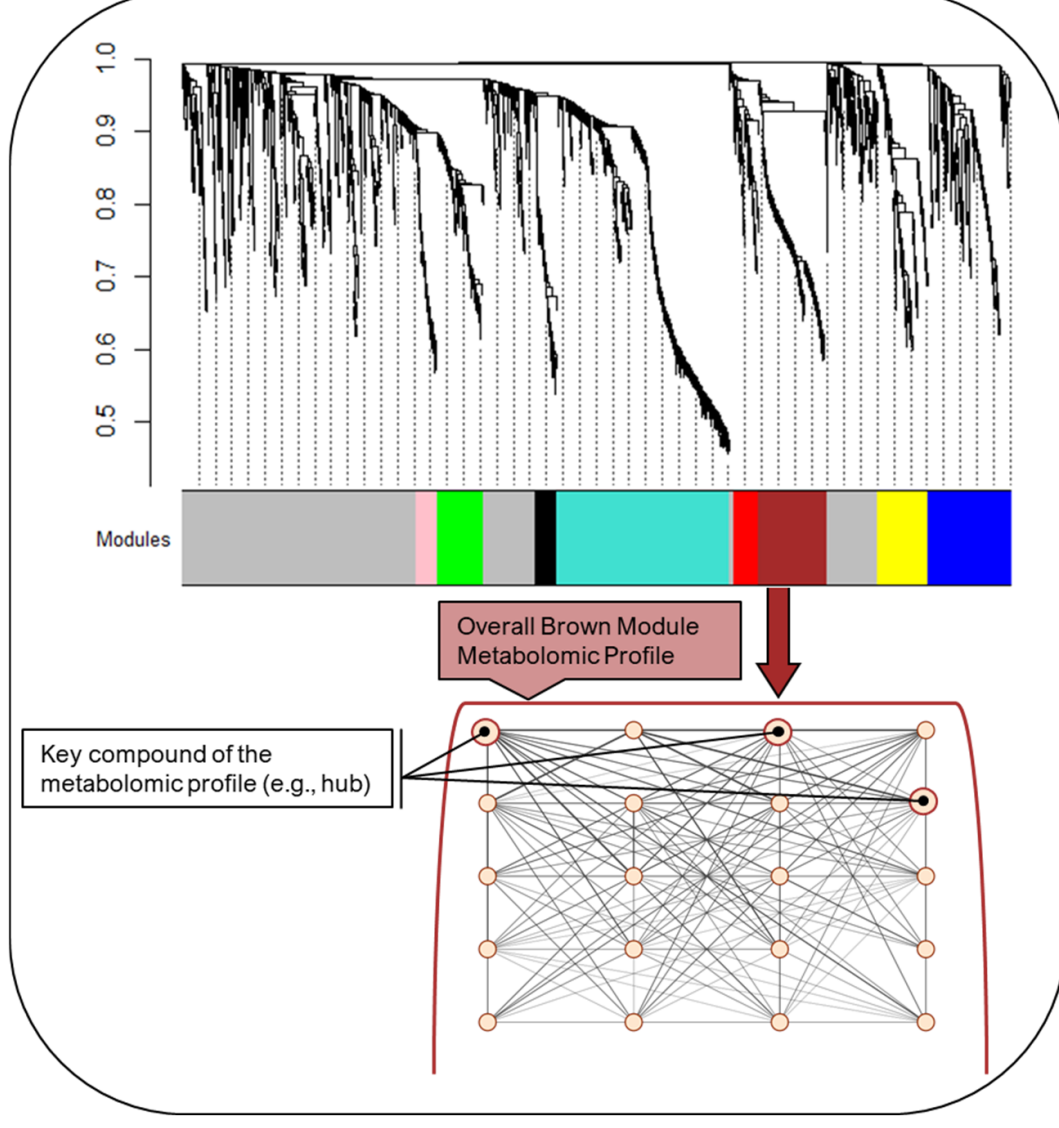
Schematic showing the global metabolomic network, as well as key modules within the network, each representing distinct metabolomic profiles. The metabolomic network and modules were derived in the Reference subgroup. The dendrogram and module colors show the hierarchical clustering, which captures the connectedness between compounds while considering their relationship to all other compounds within the network. Each color represents a module configuration characterized by highly connected compounds with few having many connections with neighboring compounds. In this figure we highlight the Brown module. The first eigenvector of the module is thought of as the average metabolomic pattern or profile captured by that module, with higher values indicating greater similarity with the module profile and lower values indicating less similarity. Within the module, the compounds that are most strongly correlated with the first eigenvector are considered to be key compounds of the profile and, accordingly, the overall module. In this figure, we show a generated example and have increased the size of the circle to indicate it is a key compound as well as increased the width of the edges to indicate it is highly connected to other compounds in the module profile.

We identified eight modules within the global metabolomic network derived in the Reference subgroup (Figure 1).

The characteristics of the modules (color scheme, size, mean connectivity, and pathway enrichment) are presented in Table 2. 

**Figure 2:**
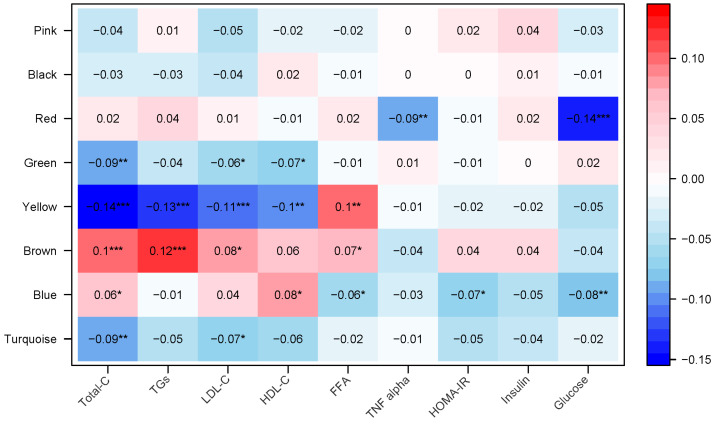
Pearson correlation between each module metabolomic profile and conventional metabolic markers. The metabolomic network and modules were derived in the Reference subgroup. Pearson correlation was used to assess the correlation between the first eigenvector of each module and the conventional metabolic biomarkers. Darker blue indicates a stronger negative correlation, darker red indicates a stronger positive correlation. * *p*-value < 0.05, ** *p*-value < 0.001, *** *p*-value < 0.0001.

The correlations with conventional metabolic biomarkers differed across some of the metabolomic network modules (Figure 2). For instance, the Brown module was positively correlated with TC, TGs, and FFAs (*p*-values <0.0001), whereas the Blue module was positively correlated with TC and HDL-C, and negatively correlated with HOMA-IR and glucose (*p* values < 0.05).

**Figure 3:**
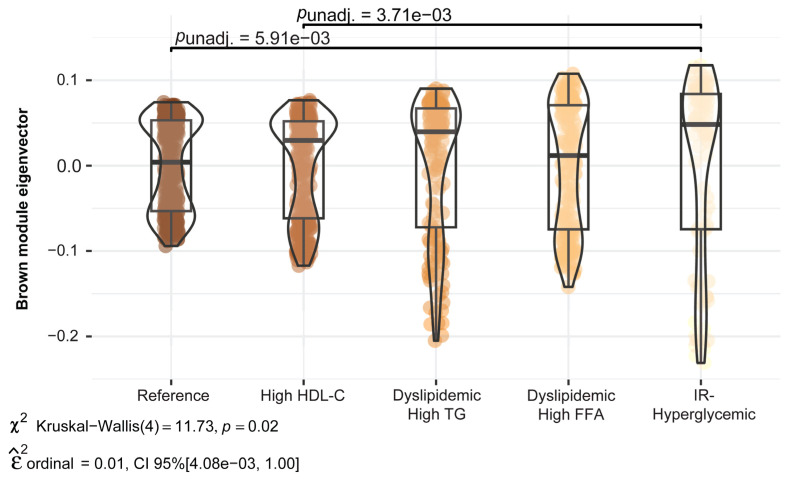
Differences in mean Brown module metabolomic profile across metabolic subgroups. The metabolomic network and modules were derived in the Reference subgroup. In this figure we show the Brown module. The first eigenvector of the module represents the average metabolomic pattern or profile captured by that module, with higher values indicating greater similarity with the module profile and lower values indicating less similarity. Pairwise test is Dunnett, with significant differences shown by a horizontal bar.

The distribution and mean differences in the score (eigenvector) for the Brown module metabolomic profile across the five metabolic subgroups are shown in Figure 3. The IR–Hyperglycemic subgroup had significantly higher mean values for this module compared to the Reference and High HDL-C subgroups (*p*-value < 0.01). The Brown module included 189 compounds and had pathway enrichment for D4- and E4-neuroprostanes, androgen and estrogen biosynthesis and metabolism, and biopterin and butyrate metabolism. The compounds that were driving differences in this profile contained phospholipids with polyunsaturated fatty acid (PUFA) tails as well as free PUFAs.

Although the metabolomic profiles of the remaining seven modules did not differ between the subgroups (Figure S1), the key compounds of the Red, Yellow, and Blue modules differed between subgroups (FDR *p*-value < 0.05). For example, the Reference subgroup had higher mean levels of the key compounds of the Blue module compared to the IR–Hyperglycemic subgroup. These compounds included lysophospholipids with a linoleate fatty acid tail, plasticizer components such as 1,2-cyclohexane dicarboxylic acid diisononyl ester (DINCH) and 2-ethylhexyl sebacate, along with an acylcarnitine and a diacylglycerol (Table S1).

### 2.3. Sensitivity Analysis

The modules exhibited high preservation across the metabolic subgroups (preservation z-summary scores ≥10) (Figure S2), indicating that these modules and the metabolites that were assigned to them were reproducible irrespective of whether the network was created in the Reference subgroup or the remaining four subgroups. We note here that good preservation signifies that the overall correlation structure of the compounds is preserved, and in the context of significant differences in module metabolomic profiles and individual compounds, this indicates that the compound levels differ between subgroups but the interrelationship between compounds is stable.

## 3. Discussion

In this study, we leveraged untargeted metabolomics profiling on fasting blood samples from 1065 women during mid-pregnancy (approximately 18 weeks gestation) and applied the weighted correlation network approach (WGCNA) to 2316 compounds across five maternal metabolic subgroups that we previously identified in this population: High HDL-C, Dyslipidemic–High TG, Dyslipidemic–High FFA, IR–Hyperglycemic, and a generally healthy Reference group [15]. A distinct metabolomic profile, referred to as the Brown module per WGCNA convention, was significantly higher among the IR–Hyperglycemic subgroup compared to the Reference group. This profile was enriched for indicators of oxidative stress response and sex hormone biosynthesis, and it was also positively correlated with TC and FFAs. Given the association of the IR–Hyperglycemic subgroup with perinatal complications and risk of offspring childhood obesity [15], these findings highlight the interplay between hyperglycemia and lipid metabolism as a pathway of developmental overnutrition [30]. Below, we discuss these findings and their implications for fetal programming.

The Brown module exhibited pathway enrichment for neuroprostane formation, DHA peroxidation, biopterin and butyrate metabolism, and androgen and estrogen biosynthesis and metabolism. The involvement of these pathways indicates upregulation of cellular processes that either drive oxidative stress or reflect its consequences. For example, omega-3 and omega-6 PUFAs modulate inflammation and oxidative stress [31,32,33], which in turn can upregulate antioxidant pathways and trigger physiological processes ranging from steroid biosynthesis to gut health and energy metabolism [34,35,36]. Inflammation has a significant role in pregnancy, ensuring an adaptive response to the physiological changes, but at excessive levels can increase risk of maternal morbidity [37]. Neuroprostanes, specifically F4-neuroprostanes, have been shown to be higher among women with pre-eclampsia [38]. High levels of oxidative stress during pregnancy can negatively impact placental function and maternal metabolic adaptations [39,40,41], and has been associated with rapid catch-up growth in offspring [40]. Key compounds responsible for differences in the eigenvector score for the Brown module were PUFAs and free PUFAs, whose peroxidation results in bioactive compounds called oxylipins, which exert both pro- and anti-inflammatory effects, depending on the enzymatic pathway [32,42]. 

Recent studies have linked higher maternal levels of oxylipins and PUFA metabolites to lower birth weight, implicating their involvement in the regulation of fetal growth [32,43]. However, in our cohort, the offspring of the IR–Hyperglycemic subgroup were at greater risk of obesity, and these differences in findings could reflect the greater impact of fetal exposure to excess fuels such as glucose, TGs, and FFAs, compared to the metabolites/compounds from the Brown module (e.g., oxidative stress and inflammation markers). Conversely, given that the traditional metabolic markers and metabolomics data were measured concurrently, these data could reflect the pathophysiology of the progression to insulin resistance and dyslipidemia as it is recognized that reactive oxygen species and inflammation are a contributor to impairment in insulin secretion and action [44]. Although the exact mechanisms are not fully understood, data indicate that oxidative stress can effect insulin signaling by disrupting insulin receptors and mitochondrial function [45]. While our prior work in the Healthy Start study using conventional metabolic biomarkers identified differences in insulin resistance and atherogenic lipid profiles between subgroups, our new data provide richer insights into novel differences in metabolic pathways involved in inflammatory and oxidative responses that are both crucial to pregnancy progression but can also occur in response to unfavorable physiologic/metabolic changes during pregnancy. Future studies could explore enhancement of antioxidant defenses or even modulating lipid levels in pregnancy for reducing in utero-derived offspring obesity risk.

While we only observed significant differences in eigenvector values for the Brown metabolomic profile with respect to the five metabolic subgroups, key compounds within the Red, Yellow, and Blue modules differed across subgroups. This indicates that the IR–Hyperglycemic subgroup differed from the Reference and High HDL-C subgroups based on the processes of oxidative stress captured by most compounds of the Brown profile, which could reflect either some of the cause or consequence of high insulin resistance in the first half of pregnancy. In contrast, only specific compounds of the Red, Yellow, and Blue modules differed between subgroups, suggesting fewer differences in the broader metabolic profiles captured by these modules. For instance, the Blue module, which was enriched for carnitine–acylcarnitine translocase activity, de novo fatty acid biosynthesis, and retinol and glycerophospholipid metabolism, had key compounds that were significantly different across subgroups. In both pregnant and non-pregnant populations short-chain acylcarnitines, which facilitate the transport of fatty acids through the mitochondrial membrane for beta oxidation, have been related to hyperglycemia (e.g., GDM and type 2 diabetes) [46,47]. We found that the Blue module was positively correlated with TC and HDL-C and inversely correlated with HOMA-IR, and thus could be capturing aspects of cholesterol metabolism that reduces lipid overload to the benefit of insulin sensitivity [48]. In addition, these differences in key compounds from the Red, Yellow, and Blue modules could reflect a range of factors that influence metabolism such as dietary patterns, lifestyle, genetics, and microbiome composition [17].

Of note, the remaining modules and their top compounds did not statistically differ across the five subgroups. The modules that were similar between subgroups (e.g., Pink, Black, Green, and Turquoise) were generally enriched for amino acid metabolism, particularly for pathways of energy metabolism and protein synthesis. The degree of consistency may reflect metabolic processes that tend to be well-conserved during pregnancy. The Healthy Start study is a general-risk pregnant population characterized by only minor gestational complications; thus, these data may suggest that among generally healthy pregnancies, the extent of metabolic overlap may be greater or equivalent to the extent of metabolic differences. Indeed, these findings align with our prior work in a separate cohort where offspring metabolomic profiles in childhood and adolescence were associated with exposure to maternal obesity or GDM, but only minimally differed depending on whether they were exposed to maternal obesity only, GDM only, or obesity and GDM [1]. Moreover, even in untargeted metabolomic studies comparing women with and without gestational complications [18,19,20,21,22,23], not all aspects of their metabolomic profiles differed, which highlight that the pathophysiology of overt complications likely involves specific metabolic pathways rather than impacts the entirety of the metabolome. 

### Study Strengths and Limitations

A key strength of this study is the large number of pregnant women with conventional metabolic biomarkers and untargeted metabolomics measured from the same fasting blood draw in the first half of pregnancy, which allowed us to gain better understanding of the metabolomic profiles. In addition, our study included general-risk pregnant women who were mostly healthy with only a few with GDM. Even among this relatively low-risk group, we were able to detect differences in metabolic pathways subgroups of pregnant women whose offspring we previously found to exhibit differential risk of future obesity [15]. However, we note that these data may be biased toward healthier pregnancies and therefore may not be generalizable to pregnant women with pre-existing chronic conditions or complicated pregnancies.

This study also has some limitations. First, we used WGCNA to characterize the metabolomic network in the Reference subgroup, which assumes that the core metabolite regulatory network remains stable, which may not always be the case, though in sensitivity analysis we found that the network was highly preserved across the different metabolic subgroups. Further, the network modules identified herein might not be identifiable in other study populations with different background characteristics, especially those at higher obstetric risk owing to pre-existing chronic conditions. Lastly, we utilized untargeted metabolomics from fasting serum, which is advantageous for uncovering broad-scale metabolic differences that provide insights into specific biological processes but provides information on relative concentrations of analytes. Future research using targeted assays for replication and validation is needed and may consider targeting bioactive analytes of dietary fatty acids such as omega-3 and omega-6.

## 4. Materials and Methods

### 4.1. Setting and Participants 

Study participants were from the Healthy Start study, a prospective pre-birth cohort of 1410 racially/ethnically diverse pregnant women enrolled from prenatal clinics at the University of Colorado Hospital between 2009–2014 [14,49]. The inclusion criteria were >15 years old, no history of stillbirth, <24 weeks gestation, singleton birth, and no pre-existing chronic disease. The study involved two in-person research visits during mid- (median 18 gestational weeks) and late (median 27 gestational weeks) pregnancy. At these visits, trained research assistants collected fasting blood samples. The final analytic sample comprised 1065 pregnant women for whom we had a sufficient volume of serum for untargeted metabolomics profiling using the mid-pregnancy sample. The women in this analysis are similar to those excluded (*n* = 345), with the exception of slightly older age (28 vs. 27 years), higher proportion of college graduates (47% vs. 33%), and slightly higher BMI (28 vs. 27 kg/m^2^). All participants provided written informed consent and the study was approved by the Colorado Multiple Institutional Review Board. Healthy Start is registered as an observational study at clinicaltrials.gov (NCT02273297).

### 4.2. Sociodemographic, Lifestyle, and Perinatal Characteristics Data

Maternal race/ethnicity (which we view as a social construct [50]), educational attainment, parity, dietary intake, and prenatal smoking status were self-reported via questionnaires [14,51,52]. We calculated maternal pre-pregnancy BMI (kg/m^2^) using pre-pregnancy weight and measured height. Gestational weight gain (GWG) was estimated by subtracting pre-pregnancy weight from the last clinically measured weight during pregnancy and categorized based on the National Academy of Medicine 2009 Guidelines [24]. All women were screened for GDM at 24–28 weeks and GDM diagnosis was abstracted from medical records and based on Carpenter–Coustan criteria [53].

### 4.3. Metabolic Subgroups Based on Conventional Metabolic Biomarkers: Laboratory Methods, Subgroup Derivation, and Analytic Approach

Laboratory methods and analysis details on the metabolic subgrouping have been published [15]. In brief, we implemented k-means clustering on seven metabolic biomarkers: glucose, insulin, total cholesterol (TC), high-density lipoprotein cholesterol (HDL-C), triglycerides (TGs), free fatty acids (FFAs), and tumor necrosis factor-α (TNFα), and two derived indices (TGs:HDL-C and the Homeostatic Model Assessment of Insulin resistance (HOMA-IR)), and retained five clusters, or “subgroups,” labeled: ‘High HDL-C’, ‘Dyslipidemic–High TG’, ‘Dyslipidemic–High FFA’, ‘IR–Hyperglycemic’, and the Reference group, which captured a favorable metabolic profile and was the largest subgroup. These seven metabolic biomarkers were chosen based on their association with important metabolic outcomes such as diabetes and hypertension [25,26,27,28,29]. We compared the distribution of maternal sociodemographic, lifestyle, and perinatal characteristics across categories of the previously derived maternal metabolic subgroups via assessment of means (SD) and a Wald test for continuous variables, and % (*N*) and a chi-squared test for categorical variables. Within each metabolic subgroup, we also assessed the prevalence of women who met the criteria for clinically relevant atherogenic dyslipidemia [25,27,29] and elevated fasting glucose [25,26,28], and sample-specific cut-offs of the 75th percentile for biomarkers measured at ~18 gestational weeks.

### 4.4. Untargeted Metabolomic Profiling: Laboratory Methods, Profile Derivation, and Analytical Approach

Metabolomics analysis was performed by the North Carolina Human Health Exposure Analysis Resource Hub funded by the National Institute of Environmental Health Sciences (NIEHS U2C ES030857). Data were acquired on a Vanquish UHPLC system coupled to a Q Exactive™ HF-X Hybrid Quadrupole-Orbitrap Mass Spectrometer (Thermo Fisher Scientific, San Jose, CA, USA). The UHPLC-HRMS data was processed by Progenesis QI (version 2.1, Waters Corporation, Milford, MA, USA). Peaks detected by UHPLC-HR-MS were identified or annotated by matching to an in-house physical standards library and public databases (NIST, HMDB) within ADAP-KDB (https://www.adap.cloud accessed on 14 November 2022), a resource for compound identifications and annotations via library matching.

We applied a data filtering process that excluded compounds if the analyte concentration in the quality control samples had a relative standard deviation >30% (high variability), if the missingness was >10% across the study samples, or if the variability across the study samples was <10th percentile of the ranked interquartile range for the compound (low-to-no variability). Prior to data analysis, we scaled and natural log (ln)-transformed all 2316 compounds following filtering. Using the filtered data, we used a weighted correlation network analysis (WGCNA) algorithm to construct a metabolomic network. Details on the WGCNA algorithm and nomenclature have been reported elsewhere [54]. Briefly, the algorithm constructs a network by first generating an adjacency matrix, which uses Pearson correlations (R^2^) to measure the connectedness between compounds. The adjacency matrix is then transformed to a topological overlap matrix which measures the connectedness between compounds considering their relationship to all other compounds within the network and subsequently performs hierarchical clustering on the topological overlap matrix based on dissimilarity. The Dynamic Tree Cut function is used to identify network modules consisting of highly correlated compounds that follow a scale-free topology, which is a type of module configuration characterized by a few compounds having many connections with neighboring compounds [54,55]. We created the global network in the Reference subgroup as it represents the healthiest women and had the largest sample size of all the subgroups (*n* = 360), and then superimposed that network onto the metabolomic data of each of the remaining four subgroups. For network creation, we used the recommended parameters of the WGCNA algorithm [54,55] and set the smallest number of compounds in each module to 30 and the least dissimilarity between modules to ≥0.20 (scale-free R^2^ > 0.80), as previously recommended [54]. A schematic of the WGCNA network is shown in Figure 1.

For statistical and quantitative analysis, we used the first eigenvector of each module as a score representing the metabolomic profile captured by that module. We first evaluated Pearson R^2^ of each metabolomic module profile in relation to the conventional metabolic biomarkers glucose, insulin, TC, HDL-C, TGs, FFAs, TNFα, and HOMA-IR. Next, we compared differences in metabolomic module profiles across the five previously identified metabolic subgroups using pairwise comparisons and a Kruskal–Wallis test to identify significant differences, with the Reference subgroup as the referent. To hone in on specific compounds within a module that were either driving the profile differences or were key to the profile, we used the compound profile membership score, which is the Pearson R^2^ between the compound concentration and the first eigenvector of the respective module. We compared differences in these individual compounds across metabolic subgroups with an ANOVA, with a Benjamini–Hochberg false discovery rate (FDR) correction, and considered an FDR *p*-value <0.05 to be statistically significant [56].

To interpret metabolic pathways associated with each module, we used the mummichog algorithm to identify the enriched pathways or functionally related compounds based on the *p*-value of the correlation (Pearson R^2^) of the compound with the first eigenvector of each module [57,58]. We used the Kyoto Encyclopedia of Genes and Genomes (KEGG) library to map the mass/charge ratio (*m*/*z*) to specific compounds and their associated pathways. We also used the North Carolina Human Health Exposure Analysis Resource Hub’s in-house library and a library of curated public databases to assign potential compound annotations for the top ten compounds based on the profile membership score in each module.

### 4.5. Sensitivity Analysis

To assess the robustness of the metabolomic network, we superimposed the metabolomic network generated in the Reference subgroup on those from the other subgroups and assessed module preservation using a quantitative measure, the preservation Z-summary score, developed for the WGCNA package [59]. The preservation Z-summary score is the aggregate of several module preservation statistics, where generally >10 indicates high preservation [60]. 

### 4.6. Missing Data

For the clinical perinatal variables, 8% of participants were missing data on GDM (*n* = 78) or GWG (*n* = 5), and among the nine conventional biomarkers, 5% of participants were missing data on at least one biomarker, which was most frequently free fatty acids (*n* = 26). We used multiple-chained equations with classification and regression trees for the imputation of missing data [29]. 

Statistical analysis was performed in R (version 4.1.2, R Foundation for Statistical Computing, Vienna, Austria)

## 5. Conclusions

In this prospective pre-birth cohort, we identified distinct metabolomic profiles in fasting blood during mid-pregnancy that captured pathways associated with DHA peroxidation, indicators of oxidative stress, and sex hormone synthesis. These profiles were markedly different between a subgroup of women previously identified as being Insulin-Resistant and Hyperglycemic and a healthy Reference group. Our findings can inform future investigations focused on the discovery of novel markers for pregnancy risk stratification and unraveling mechanisms related to the developmental origins of metabolic disease risk by linking metabolomic profiles to long-term maternal and offspring health outcomes.

## Figures and Tables

**Table 1:** Characteristics of 1065 pregnant women in the Healthy Start study, overall and by metabolic subgroup membership.

	Full Sample	Reference	High HDL-C	Dyslipidemic–High TG	Dyslipidemic–High FFA	IR–Hyperglycemic	
Maternal Characteristics:	(*n* = 1065)	(*n* = 360)	(*n* = 289)	(*n* = 149)	(*n* = 180)	(*n* = 87)	*p*-Value
Age, years; mean ± SD	28.1 ± 6.2	27.9 ± 6.5	29.1 ± 5.7	28.6 ± 5.9	27.1 ± 6.3	26.2 ± 6.3	<0.001
race/ethnicity (%, *n*)							<0.001
Hispanic	23.4 (249)	59.4 (214)	65.1 (188)	55.7 (83)	43.3 (78)	32.2 (28)	
Non-Hispanic Black	15.1 (161)	19.4 (70)	11.8 (34)	4.7 (7)	18.9 (34)	18.4 (16)	
Non-Hispanic White	55.5 (591)	16.1 (58)	17.3 (50)	30.9 (46)	33.3 (60)	40.2 (35)	
Non-Hispanic Other ^a^	6.0 (64)	5.0 (18)	5.9 (17)	8.7 (13)	4.4 (8)	9.2 (8)	
Education (%, *n*)							<0.001
High school or less	30.8 (328)	30.3 (109)	18.0 (52)	37.6 (56)	36.7 (66)	51.7 (45)	
Some college/assoc. degree	22.4 (238)	17.2 (62)	23.5 (68)	25.5 (38)	26.1 (47)	26.4 (23)	
College graduate	23.9 (255)	23.3 (84)	28.7 (83)	22.8 (34)	22.2 (40)	16.1 (14)	
Graduate degree	22.9 (244)	29.2 (105)	29.8 (86)	14.1 (21)	15.0 (27)	5.8 (5)	
Nulliparous (%, *n*)	48.5 (516)	47.5 (171)	57.1 (165)	40.3 (60)	47.2 (85)	40.2 (35)	0.002
Smoked during pregnancy (%, *n*)	8.1 (87)	9.2 (33)	3.5 (10)	13.4 (20)	8.3 (15)	10.3 (9)	0.002
Pre-pregnancy BMI; mean ± SD	25.5 ± 6.0	23.8 ± 4.5	23.8 ± 4.4	27.1 ± 5.2	26.5 ± 6.6	33.1 ± 8.5	<0.001
Pre-pregnancy BMI ≥30.0 kg/m^2^ (%, *n*)	18.5 (196)	10.3 (37)	7.6 (22)	28.2 (42)	24.7 (44)	58.6 (51)	<0.001
Gestational diabetes mellitus (%, *n*)	4.4 (43)	1.2 (4)	2.6 (7)	6.0 (8)	6.0 (10)	17.3 (14)	<0.001
Gestational weight gain (%, *n*)							0.029
Insufficient	23.5 (249)	25.4 (91)	19.8 (57)	25.5 (38)	23.6 (42)	24.1 (21)	
Adequate	29.2 (309)	31.3 (112)	29.9 (86)	30.2 (45)	27.0 (48)	20.7 (18)	
Excessive	47.4 (502)	43.3 (155)	50.4 (145)	44.3 (66)	49.4 (88)	55.2 (48)	
**Biomarkers ~18 gestational** **weeks** (%, *n*)							
Glucose ≥ 95 mg/dL	1.5 (16)	0.8 (3)	0.4 (1)	0.7 (1)	0.0 (0)	12.6 (11)	<0.001
Insulin ≥ 25 uIU/mL	8.4 (88)	0.3 (1)	0.4 (1)	8.2 (12)	2.3 (4)	81.4 (70)	<0.001
HOMA-IR ≥ 2.9	24.5 (255)	12.6 (44)	10.1 (29)	44.5 (65)	18.3 (32)	100.0 (85)	<0.001
TGs:HDL-C ≥ 2.5	27.0 (283)	9.1 (32)	5.2 (15)	95.9 (139)	28.8 (51)	52.9 (46)	<0.001
TGs ≥ 150 mg/dL	23.8 (249)	3.7 (13)	15.9 (46)	90.3 (131)	13.0 (23)	41.4 (36)	<0.001
Total-C ≥ 200 mg/dL	31.2 (327)	4.0 (14)	72.7 (210)	46.2 (67)	11.3 (20)	18.4 (16)	<0.001
HDL-C ≤ 50 mg/dL	20.3 (213)	19.4 (68)	0.0 (0)	43.5 (63)	27.7 (49)	37.9 (33)	<0.001
FFAs ≥ 472 µEq/L	24.4 (253)	0.3 (1)	13.2 (38)	27.6 (40)	81.7 (143)	35.6 (31)	<0.001
TNF-α ≥ 1.36 pg/mL	25.3 (266)	27.5 (97)	22.8 (66)	26.2 (38)	26.1 (46)	21.8 (19)	0.646

^a^ Non-Hispanic Other: due to low cell sizes for some of the categories, we combined American Indian or Alaska Native, Asian, Native Hawaiian or Pacific Islander, and >1 race into a single category of “non-Hispanic Other”. Gestational weight gain categories classified according to the Institute of Medicine 2009 guidelines [24]. Biomarker cut-offs: glucose, TGs, HDL-C [25,26,27]; insulin, Total-C [25]; HOMA-IR [26,28]; TGs:HDL-C [29]; FFAs, TNF-α (>75th percentile in the full sample). Abbreviations: BMI, body mass index; FFAs, free fatty acids; HOMA-IR, homeostatic model of insulin resistance, HDL-C, high-density lipoprotein cholesterol; IR, insulin-resistant; TGs, triglycerides; TNF-α, tumor necrosis factor-α; Total-C, total cholesterol.

**Table 2:** Metabolomic network modules (labeled by color), size (# of features within a module), and pathway enrichment.

Module Color Label	Module Size (# of Features)	Mean Connectivity	Pathway Enrichment
Pink	59	21.2	Pyruvate metabolism Glycine, serine, alanine and threonine metabolism
Black	60	17.7	Tyrosine metabolism Aspartate and asparagine metabolism Glycine, serine, alanine and threonine metabolism
* Red	69	7.3	Urea cycle/amino group metabolism Aspartate and asparagine metabolism
Green	130	24.9	Squalene and cholesterol biosynthesis Hexose phosphorylation Fatty acid metabolism
* Yellow	138	14.5	Bile acid biosynthesis Linoleate metabolism Fatty acid activation and metabolism De novo fatty acid biosynthesis Omega-3 fatty acid metabolism C21-steroid hormone biosynthesis and metabolism
* Brown	189	30.5	Androgen and estrogen biosynthesis and metabolism C21-steroid hormone biosynthesis and metabolism Cytochrome P450 enzymes D4 & E4-neuroprostanes formation Biopterin metabolism Butyrate metabolism
* Blue	231	8.9	Carnitine shuttle De novo fatty acid biosynthesis Vitamin A (retinol) metabolism Glycerophospholipid metabolism
Turquoise	400	50.1	Tryptophan metabolism Tyrosine metabolism Aspartate and asparagine metabolism

Table 2 includes each module within the metabolomic network, the module color label, size (e.g., number of features), mean connectivity, and the metabolic pathways that were enriched. Mean connectivity (range is 0 to n—1, where *n* = module size) of a module is a metric of intramodular connectivities of all the compounds within that module and gives you a sense of how tightly connected the compounds are within the module. To identify the pathways and compounds that the modules represented, we applied the mummichog algorithm, which uses an over-representation analysis to identify the enriched pathways or functionally related compounds based on the significance level of each feature. The KEGG (Kyoto Encyclopedia of Genes and Genomes) library reference was used to map mass/charge (*m*/*z*) features to potential compounds and their associated pathways. The significance level of each compound was the *p*-value obtained during module assignment, which the mummichog algorithm used to rank the importance of each feature within the given module. All compounds were used as the refence for enrichment. * Indicates this module also had key compounds that were significantly different between metabolic subgroups which are shown in Table S1.

## Data Availability

Metadata for the Healthy Start study are available upon reasonable request to the PIs. Untargeted metabolomics data are available at the NIH Common Fund’s National Metabolomics Data Repository (NMDR) website, the Metabolomics Workbench, https://www.metabolomicsworkbench.org accessed on 14 November 2022, where it has been assigned Project ID (PR001473). The data can be accessed directly via the Project DOI: (https://doi.org/10.21228/M8B40C accessed on 14 November 2022). This work is supported by the Metabolomics Workbench/National Metabolomics Data Repository (NMDR) (grant number U2C-DK119886), Common Fund Data Ecosystem (CFDE) (grant number 3OT2OD030544), and Metabolomics Consortium Coordinating Center (M3C) (grant number 1U2C-DK119889).

## Supplementary Materials

The following supporting information can be downloaded at https://www.mdpi.com/article/10.3390/ijms25147620/s1.
