# Supplementary material for: Maternal Serum Metabolomics in Mid-Pregnancy Identifies Lipid Pathways as a Key Link to Offspring Obesity in Early Childhood
Source: Int J Mol Sci. 2024 Jul 11;25(14):7620. doi: 10.3390/ijms25147620 (PMC11276882; doi:10.3390/ijms25147620)
Supplement: Supplementary file 1 [file ijms-25-07620-s001.zip › eMaterial_Metabolomics Subgroups submitted.pdf]

Title: Metabolic Subgroups in Pregnancy and a Description of Associated Untargeted Metabolomic Profiles
Supplementary Material

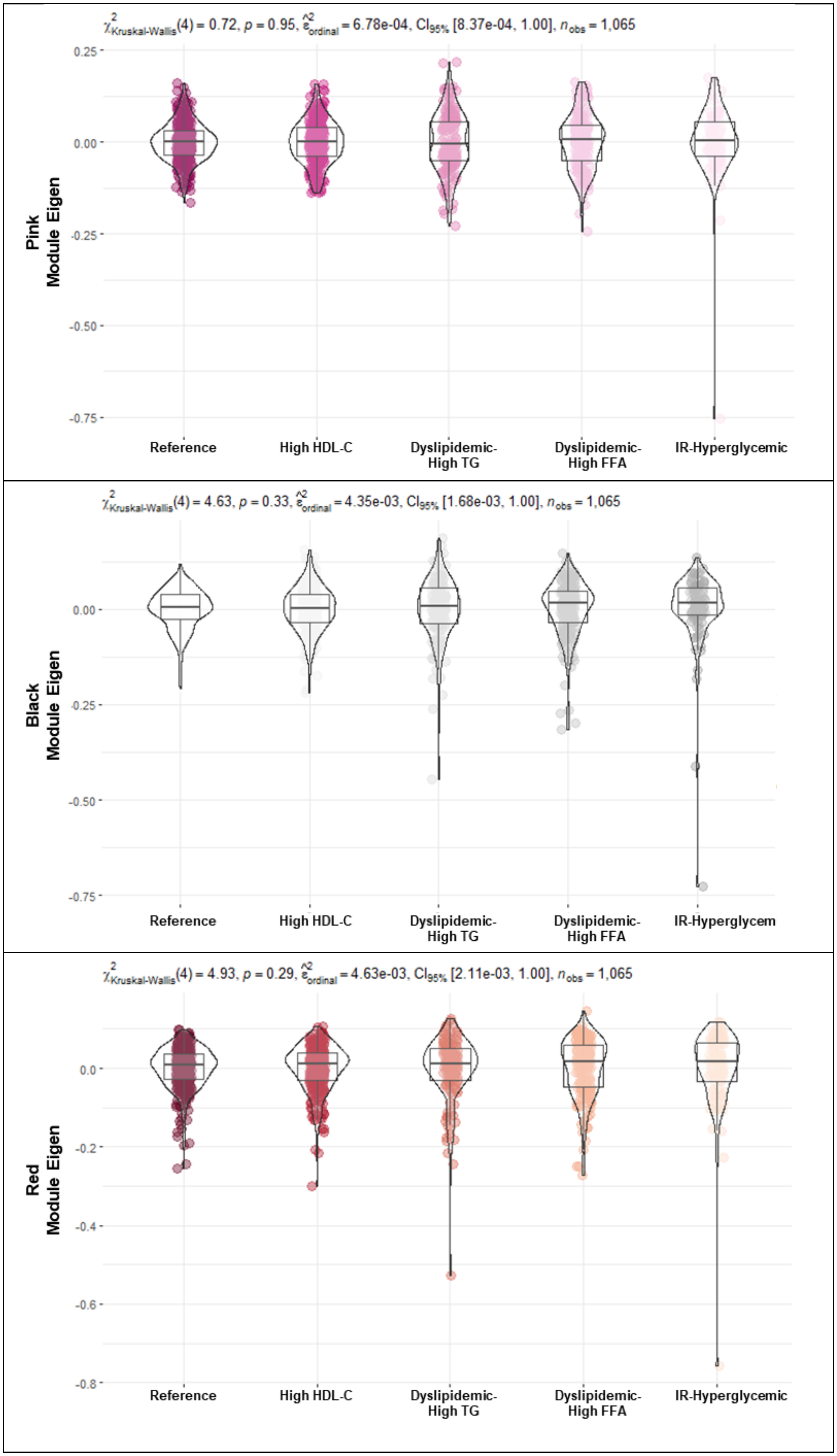

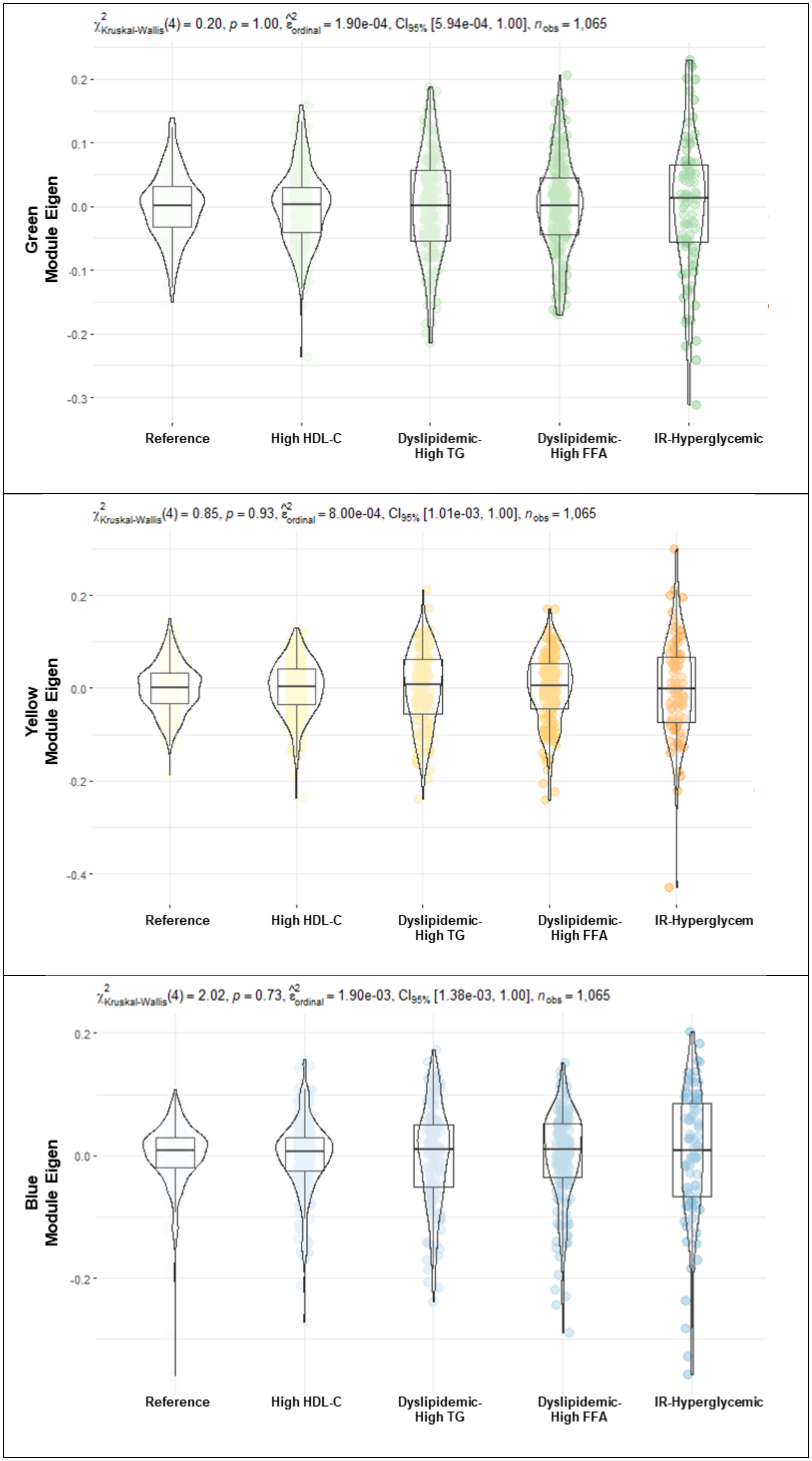

$\chi^2_{Kruskal-Wallis}(4) = 0.20$, $p = 1.00$, $\hat{\epsilon}^2_{ordinal} = 1.90e-04$, $CI_{95\%}$ [5.94e-04, 1.00], $n_{obs} = 1,065$
Green Module Eigen
0.2
0.1
0.0
-0.1
-0.2
-0.3
Reference
High HDL-C
Dyslipidemic-High TG
Dyslipidemic-High FFA
IR-Hyperglycemic
$\chi^2_{Kruskal-Wallis}(4) = 0.85$, $p = 0.93$, $\hat{\epsilon}^2_{ordinal} = 8.00e-04$, $CI_{95\%}$ [1.01e-03, 1.00], $n_{obs} = 1,065$
Yellow Module Eigen
0.2
0.0
-0.2
-0.4
Reference
High HDL-C
Dyslipidemic-High TG
Dyslipidemic-High FFA
IR-Hyperglycem
$\chi^2_{Kruskal-Wallis}(4) = 2.02$, $p = 0.73$, $\hat{\epsilon}^2_{ordinal} = 1.90e-03$, $CI_{95\%}$ [1.38e-03, 1.00], $n_{obs} = 1,065$
Blue Module Eigen
0.2
0.0
-0.2
Reference
High HDL-C
Dyslipidemic-High TG
Dyslipidemic-High FFA
IR-Hyperglycemic

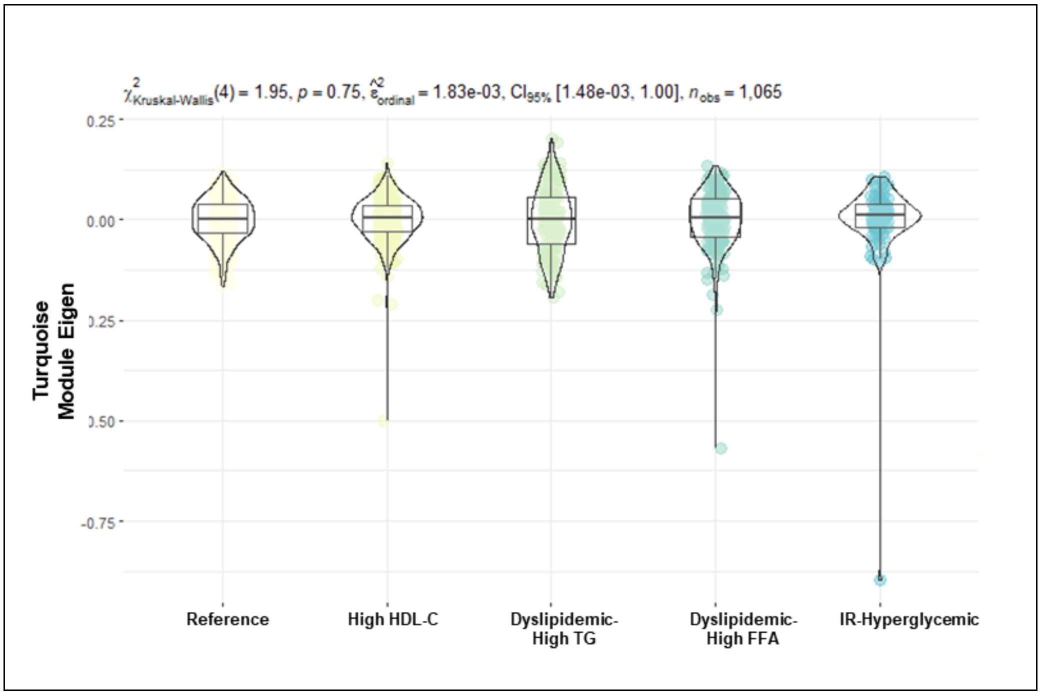

Figure S1. Metabolomic Profile of Each Module by Metabolic Subgroup

For each network module, the first eigenvector of a given module and can be considered representative of that module's metabolomic profile. Data are the mean values of the metabolomic profile for each module for the individuals in the different subgroups. Mean differences were tested using pairwise comparisons and a Kruskal-Wallis test to identify significant differences. All statistical tests for mean differences between subgroups for the metabolomic profiles of Pink, Black, Red, Green, Yellow, Blue, Turquoise modules were not significant.

Table S1. Differences in Means of Module Central Compounds Between Metabolic Subgroups

This table is included as a supplementary Excel "Additional File 2 eTable1.xlsx"

The top 10 compounds of each network module are considered drivers/central because they are the compounds that are highly correlated with the overall metabolomic profile (eigenvector) of the module and are highly connected to other compounds within the module. Means of each central compound were compared across metabolic subgroups and a false discovery rate correction using the Benjamini and Hochberg procedure was applied. Q-values <0.05 were considered significantly different between subgroups.

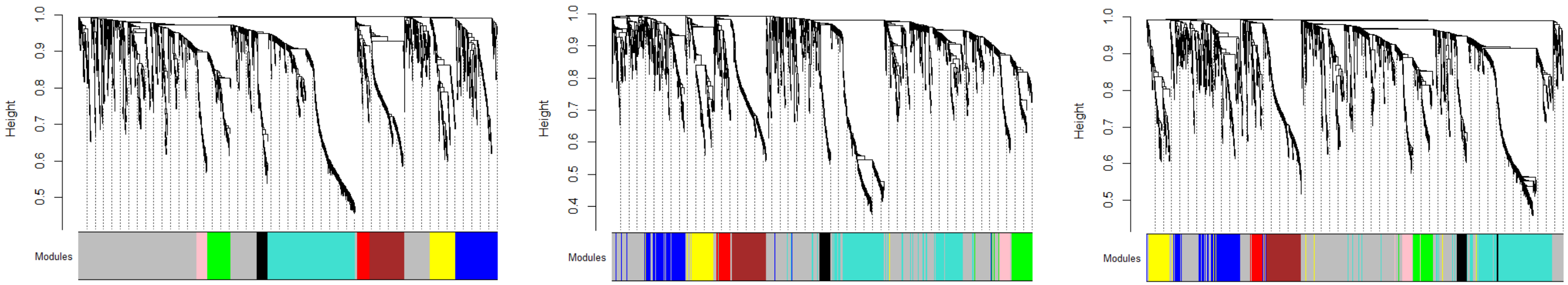

Reference network and module label	High HDL-C module preservation z-score	Dyslipidemic-High TG module preservation z-score
Pink	23.19	18.51
Black	19.23	15.60
Red	17.43	14.67
Green	28.97	25.29
Yellow	23.32	19.33
Brown	31.01	27.28
Blue	16.48	16.72
Turquoise	40.28	42.20

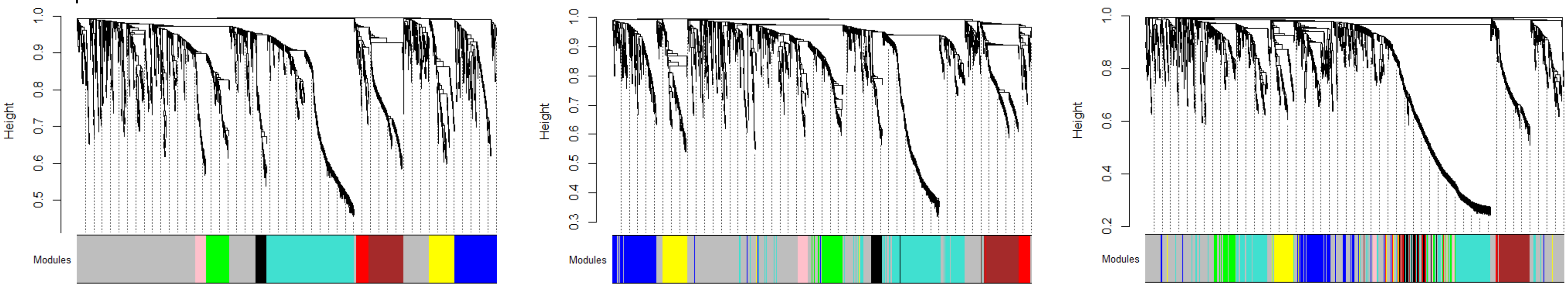

Reference network and module label	Dyslipidemic-High FFA module preservation z-score	IR-Hyperglycemic module preservation z-score
Pink	18.57	9.99
Black	16.71	14.06
Red	15.23	10.79
Green	26.01	12.18
Yellow	24.11	15.43
Brown	27.60	20.74
Blue	17.74	16.21
Turquoise	42.22	24.05

Figure S2. Qualitative and Quantitative Measures of Metabolomic Network Preservation Across Different Metabolic Subgroups. The metabolomic network was derived in the Reference group and then superimposed onto the compounds of each respective metabolic subgroup. The Reference network and module labels are shown in the right-hand column and are repeated in the 1st and 2nd row to facilitate comparisons. The dendrograms and module colors show a qualitative assessment of the preservation of the Reference metabolomic network in each of the metabolic subgroups. The module preservation z-scores below each dendrogram are a quantitative assessment of the preservation of the network and modules in each of the metabolic subgroups. Overall, the metabolomic network is preserved across different metabolic subgroups, with a preservation score >10 indicating good preservation of the module.
